# Preservation of myocardial contractile function by aminoguanidine, a nitric oxide synthase inhibitors, in a rat model of hemorrhagic shock

**DOI:** 10.12669/pjms.296.3717

**Published:** 2013

**Authors:** Mona Soliman

**Affiliations:** 1 Dr. Mona Soliman, Associate Professor, Department of Physiology, College of Medicine, King Saud University, P.O. Box. 2925 (29), Riyadh 1146, Saudi Arabia.

**Keywords:** Aminoguanidine, Contractility, Hemorrhage, Shock

## Abstract

***Background: ***Myocardial contractile dysfunction plays a major role in the outcome of trauma patients. Nitric oxide (NO) has been shown to increase following haemorrhagic shock. Peroxynitrite which is produced by the reaction of NO with reactive oxygen species leads to nitrosative stress mediated organ injury and myocardial contractile dysfunction. Aminoguanidine is a selective inhibitor of inducible nitric oxide synthase (iNOS).

***Objectives:*** The aim of this study was to determine the protective effects of inhibiting the production of NO using aminoguanidine (AG) on myocardial contractility, following hemorrhagic shock and resuscitation in rats.

***Methods:*** Male Sprague-Dawley rats were assigned to 3 experimental groups (n = 6 per group):1) Normotensive rats (N), 2) Hemorrhagic shock rats (HS), and 3) Hemorrhagic shock rats treated with AG 60 mg/kg AG intra-arterially (HS-AG). Rats were hemorrhaged over 60 minutes to reach a mean arterial blood pressure of 40 mmHg. Rats were treated with 1 ml of 60 mg/Kg AG intra-arterially after 60 minutes haemorrhagic shock,. Resuscitation was performed in vivo by the reinfusion of the shed blood for 30 minutes to restore normo-tension. Hearts were harvested and *ex vivo* perfused in the Langendorff System and myocardial function was determined by measuring the left ventricular end diastolic pressure (LVEDP) and left ventricular systolic pressure (LVSP). Left ventricular generated pressure (LVGP) and + dP/dt max was calculated.

***Results:*** Hemorrhagic shock rats treated with AG exhibited a significant increase in left ventricular generated pressure LVGP (137.1 ± 9.4 mmHg) and + dP/dtmax (589.6 ± 110.7 mmHg/sec) compared with the untreated group (44.43 ± 20.18 mmHg, 289.8 ± 25.0 mmHg/sec).

***Conclusion:*** Treatment with AG protects the myocardium from post-resuscitation myocardial dysfunction.

## INTRODUCTION

Despite advances in the management of trauma victims, myocardial dysfunction and multiple organ failure remain leading causes of death.^1^ The specific mechanisms involved in the pathophysiology of hemorrhage have not been completely defined. To improve the resuscitation strategies, the precise mechanisms responsible for myocardial injury and subsequent cellular dysfunction and organ failure need to be highlighted.

Cardiovascular adaptation to hemorrhagic shock is modulated by different circulating endocrine and local paracrine factors such as nitric oxide (NO).^2^ Studies have shown that hemorrhagic shock leads to enhanced production of NO as well as increased tissue NO synthase (NOS) activity.^3^^,^^4^

NO is produced via three unique synthases (NOS): endothelial (eNOS), neuronal (nNOS) and (iNOS) produced primarily after stimulation. Inducible (iNOS) is elevated after hemorrhage.^3^^,^^4^ Hemorrhagic shock decreases eNOS activity.^5^ However, the role of NOS in hemorrhagic shock and its contribution to the prevention or induction of myocardial dysfunction and multiple organ failure remains elusive.

Inhibition of iNOS with the selective inhibitor N^6^- (iminoethyl)-L-lysine (l-NIL) resulted in marked reduction of liver injury following hemorrhagic shock.^6^ Inhibition of nitric oxide with N (G)-Nitro-L-arginine methyl ester (L-NAME) prevents heart rate acceleration^7^ and increases the survival rate of rats following hemorrhagic shock.^8^

Aminoguanidine (AG) is a more potent inhibitor of iNOS.^9^ Studies have shown that AG attenuates delayed circulatory failure following endotoxic shock in rats and improves survival.^10^ AG and L-NAME improve the survival rate following hemorrhagic shock and reduce microscopic injuries.^11^ However, the cardioprotective effects of NOS inhibition have not been investigated. 

In the present study, we evaluated the effect of AG, a selective inhibitor of iNOS, on myocardial contractility following *in vivo* resuscitation of hemorrhagic shock in rats.

## METHODS

***Animal Preparation: ***This study was approved by the *National Plan for Sciences and Technologies*, *King Saud University*. The study was approved by the Ethical Committee at the National Plan for Sciences and Technologies, King Saud University.

**Fig.1:**
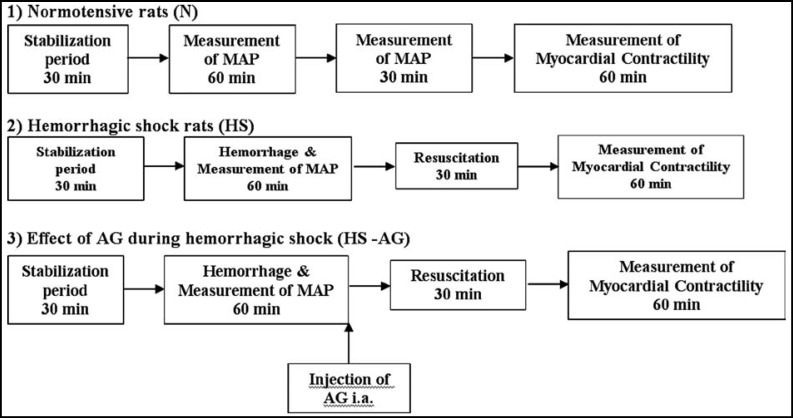
Experimental protocol. The rats were assigned to 3 groups:1) normotensive , 2) hemorrhage and 3) hemorrhage treated with AG (n = 6 per group). Rats were hemorrhaged over 60 min to reach a mean arterial blood pressure of 40 mmHg. After a 60 min hemorrhagic shock, the rats were either treated or not by injection of 1ml of (60 mg/kg) AG intra-arterially. Resuscitation was performed *in vivo *by reinfusion of the shed blood to restore normo- tension for 30 min

Male Sprague-Dawley rats weighing 300-350g were used. Rats were injected intra-peritonealy (i.p.) with 2000 I.U of heparin sodium 15 minutes prior to anesthesia. The rats were anesthetized using 125mg/kg urethane intra-peritoneally. The left carotid artery was cannulated, and a three-way stopcock was attached in-line for monitoring the mean arterial blood pressure (MABP) using a blood pressure transducer. The animals were allowed to stabilize for a period of 30 minutes. The animals were assigned to the following 3 experimental groups (n = 6 per group): 1) Normotensive rats (N), 2) Hemorrhagic shock rats (HS), and 3) Hemorrhagic shock rats treated with AG (HS-AG) (Fig. 1). After 60 minutes hemorrhagic shock, rats were treated or not by injection of 1ml of 60 mg/Kg AG intra-arterially. A pilot experiment was performed in normal control rats without a hemorrhagic insult without carotid artery cannulation to exclude any effects of carotid artery cannulation on contractility. 

***Hemorrhagic Shock: ***After a stabilization period of 30 minutes, hemorrhagic shock was induced. The rats were hemorrhaged using a reservoir (a 10 ml syringe) that was connected to the arterial (carotid artery) three way stopcocks. The hemorrhage was induced by opening the stopcock and aspirating gently and gradually with the syringe. Blood was aspirated at a rate of 1 ml/min. Blood was continuously withdrawn or re-infused to maintain MABP of approximately 35-40 mmHg. With the exception of inducing the hemorrhage, the surgical procedure was the same for the normotensive group. 

The rats were resuscitated *in vivo* by reinfusion of the shed blood to restore normotension, and the MABP was monitored for 30 minutes. 

Aminoguanidine was obtained from Sigma (Sigma, St Louis, MO). The drug was dissolved in a 0.9% sodium chloride solution (Sigma).

***Mean Arterial Blood Pressure: ***The MABP was monitored for the 60- minutes duration of hemorrhagic shock and for 30 min after resuscitation.

***Experimental Protocols***

Three experimental groups (n = 6) were assigned for the study (Fig.1):

1. **Normotensive rats (N).** The rats underwent the same surgical preparation and continuous blood pressure measurements were obtained for the 120- minutes experimental period.
2. **Hemorrhagic shock rats (HS).** After a 30-min stabilization period, the rats were hemorrhaged to 40 mmHg for 60 min. The rats were then resuscitated and monitored for 30 minutes.
3. **Effect of aminoguanidine (AG) during hemorrhagic shock (HS-AG).** After a 30 minutes stabilization period, the rats were hemorrhaged to 40 mmHg for 60 min. AG was injected intra-arterially. The rats were then resuscitated and monitored for 30 min.

***Harvest and Ex vivo Perfusion of Hearts: ***Hearts were harvested and perfused *ex vivo* for hemodynamic measurements using the Langendorff system. The hearts were perfused with a normal physiological buffer KHB solution, described below, for 60 minutes.

A sub-xiphoid transverse incision was made in the abdomen and extended superiorly along both mid-axillary lines. The diaphragm was carefully transected along its ventral margin and the ventral rib cage was lifted revealing the beating heart. The hearts were then excised quickly and placed into ice-cold physiologic saline to arrest the heart. The hearts were attached by the aorta to the proper size cannula in the Langendorff system. The hearts were perfused with non-circulating Krebs-Henseleit- bicarbonate (KHB) buffer consisting of the following (in mM): sodium chloride, 118; calcium chloride, 1.25; potassium chloride, 4.7; sodium bicarbonate, 21; magnesium sulfate, 1.2; glucose, 11; potassium biphosphate, 1.2; and EDTA, 0.5. The perfusate temperature was maintained at 37^ O^ C. The perfusate was gassed with a mixture of 95% O_2 _+ 5% CO_2 _at a pH of 7.4 for the duration of the experiment.

***Hemodynamic measurements:*** At the end of the experimental period the hearts were harvested and perfused using the Langendorff Apparatus for 60 minutes. The myocardial function was determined with a saline-filled cellophane balloon-tipped catheter that was inserted into the left ventricle via the mitral valve and was used to measure the LV pressure. The balloon was inflated to maintain an end diastolic pressure at 5 mmHg by inflating the intraventricular balloon with 0.4-0.5 ml saline at the start of the perfusion. After that the LVEDP was recorded, and no more adjustments were made in the balloon volume. The LV and perfusion pressures were measured using transducers placed at the levels of the heart and aorta. The hearts were stimulated electrically at 300 beats per minute using an electrical stimulator (6020 Stimulator from Harvard Apparatus). The frequency of the pulses was adjusted to 5 Hz, the pulse width, which is the duration of the stimulation pulses was 1 mS and the pulse amplitude was 5-7 Volts. Pacing voltage iwas determined as a set percentage (normally 110-150%) above the voltage required for the hearts to capture (pace). The perfusion pressure was maintained at 50 mmHg. The left ventricular end diastolic pressure (LVEDP) was maintained at 5 mmHg. LVEDP, the left ventricular peak systolic pressure (LVPSP), and the coronary perfusion pressure (PP) were recorded. Left ventricular generated pressure (LVGP) was calculated as the difference between the left ventricular peak systolic pressure (LVPSP) and left ventricular end diastolic pressure (LVEDP); (LVGP=LVPSP-LVEDP). The left ventricular + dP/dt, which is an index of left ventricular contractility, was calculated.

***Statistical Analysis: ***All data was initially analyzed using Bartlett’s test for homogeneity. The data were analyzed using analysis of variance (ANOVA). The means were analyzed using Duncan’s test and was considered significant when the “p” value was less than or equal to 0.05. The data was expressed as the means ± SEM.

## RESULTS

The animals were subjected to hemorrhagic shock to lower the MABP to the desired level of hypotension (35-40 mmHg). The total volume of blood withdrawn was 15 ± 2.3 mL/kg body weight. There was no significant difference in the amount of blood withdrawn among the groups of animals subjected to hemorrhagic shock.

**Fig.2:**
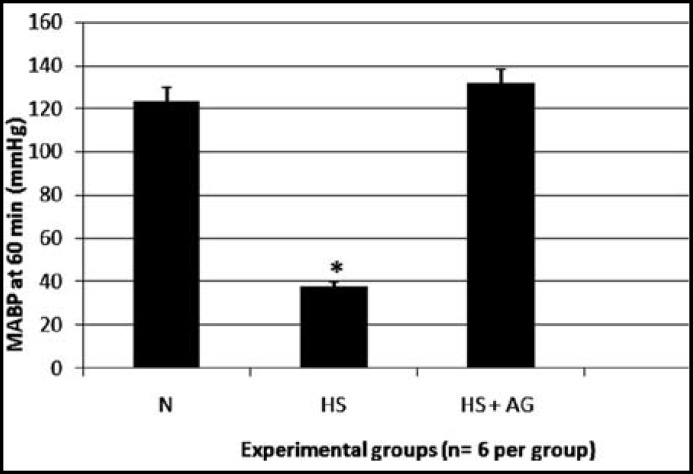
Effects of *in vivo* treatment with AG on the MABP in rats. Recording of the arterial blood pressure after one- hour hemorrhages and 30 min of resuscitation in the normotensive group (N), hemorrhage group not resuscitated without AG (HS), hemorrhage group resuscitated without AG (HS) and hemorrhage group resuscitated with AG (HS-AG). * represents p< 0.05 between the treated and non-treated hemorrhagic shock resuscitated groups

**Fig.3:**
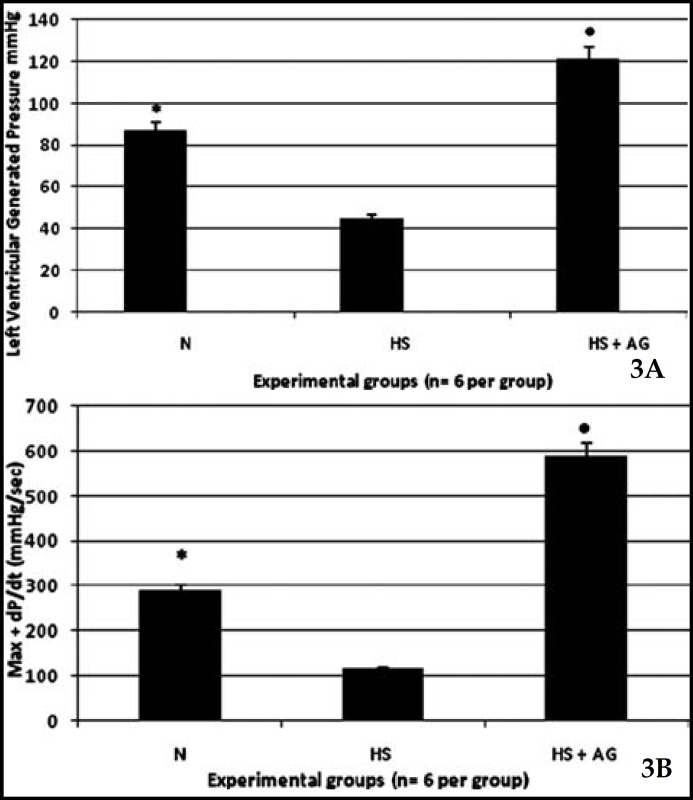
Effect of AG treatment before resuscitation of hemorrhagic shock on left ventricular function. (*a)* Left ventricular generated pressure (LVGP) in the normotensive (N), hemorrhagic shock (HS) and hemorrhagic shock + AG (HS-AG) and (*b*) positive change in pressure over time (+ dP/dt) (n= 6 per group). In *A and B*, the LVGP and + dP/dt were significantly improved in AG-treated rats compared to untreated rats. All the values are the means ± SD. * represents p< 0.05 versus hemorrhagic shock group, • represents p<0.05 versus the hemorrhagic shock group (n= 6 per group).

***Mean Arterial Blood Pressure: ***The normotensive rats maintained MABP at approximately 123.7 ± 4.8 mmHg, whereas the hemorrhagic shocked rats exhibited significantly lower levels of MABP at approximately 38.2 ± 1.3 mmHg. In hemorrhagic shocked animals, intra-arterial administration of AG (60 mg/kg) did not cause a significant difference in the MABP at the end of the resuscitation compared with the normotensive and the hemorrhagic shock group (Fig.2).

***AG prevented myocardial contractile dysfunction after hemorrhagic shock: ***The measurement of myocardial contractile function in the isolated hearts that was measured using the Langendorff system after treatment with AG and resuscitation of the hemorrhagic shocked animals showed improved myocardial function compared with the HS group (Fig.3). The left ventricular generated pressure was significantly lower in the hemorrhagic shock group (44.4 ± 17.9 mmHg) compared with the normotensive group (87.0 ± 15.8 mmHg) (Fig.3a). The left ventricular generated pressure was significantly higher in animals treated with AG (137.05 ± 9.39 mmHg) than in hemorrhage non-treated animals (p< 0.05) (Fig.3a). The left ventricular +dP/dtmax was significantly lower in the hemorrhagic shock group (115.05 ± 35.7 mmHg/s) than in the normotensive group (289.83 ± 25.04 mmHg/s) (Fig.3b). The +dP/dtmax was significantly higher in the animals treated with AG (589.6 ± 25. 04 mmHg) than in hemorrhage non-treated animals (p< 0.05) (Fig.3b).

## DISCUSSION

The present study demonstrated that aminoguanidine, a selective inhibitor of iNOS, protect against myocardial contractile dysfunction after resuscitation from hemorrhagic shock in rats. It also showed that hemorrhagic shock resulted in myocardial contractile dysfunction following hemorrhagic shock and resuscitation, which suggested that myocardial dysfunction was due to, at least in part, to the increased production of NO. 

Previous studies have shown that hemorrhagic shock has been associated with the over production of iNOS which leads to increased production of NO.^12^^,^^13^ Nitric oxide may mediate the pathophysiology of hemorrhagic shock.^4^^,^^11^ Regulation of NO production by NOS inhibitors might provide a new aspect in the treatment of hemorrhagic shock. The present study demonstrates that AG, a selective inhibitor of iNOS, improved myocardial contractility following resuscitation in a rat model of hemorrhagic shock. The improved myocardial contractility is likely caused by the inhibition of iNOS by AG.

Previous studies have shown the beneficial effects of AG in hemorrhagic shock models on the survival rate and heart rate,^8^^,^^11^ including an increase in cardiac output,^14^^,^^15^ improvement in renal blood flow and glomerular filtration rate.^16^ Hua and Moochhala investigated the influence of AG on the survival rat in a rat model of hemorrhagic shock.^11^ The study demonstrate that AG increased the survival rate following hemorrhagic shock, and the effect was dose dependent. The study also showed that pronounced macroscopic and microscopic multiple organ injuries occurred following hemorrhagic shock in rat, while AG significantly reduced the incidence of organ damage.

The study proved that hemorrhagic shock and resuscitation result in myocardial contractile dysfunction and that AG protected the heart against postresuscitation myocardial dysfunction. One probable mechanism is enhanced NO synthesis. NO synthase inhibitors such as AG are potential therapeutic agents in the experimental therapy of hemorrhagic shock. NO synthase inhibitors protect against post-resuscitation myocardial dysfunction in rats subjected to hemorrhagic shock and resuscitation. Further studies are needed to clarify the mechanism of action, but our hypothesis isthat AG inhibits the excessive NO formation that occurs during hemorrhagic shock.

In our study, the administration of AG did not alter MABP compared with the untreated group. NO is a potent vasodilation factor that has been implicated in the pathogenesis of various shock forms including hemorrhage.^17^ Our finding is similar to Hua and Moochhala^11^ who found that administration of AG did not alter blood pressure in a rat model of hemorrhagic shock. Their hypothesis in their study was that AG inhibit excessive NO formation that occur during hemorrhagic shock and that may help in maintaining the vascular reactivity and thus restoring the blood pressure, and preventing severe organ injury and mortality associated with vascular impairment.

## CONCLUSIONS

The present study has demonstrated the beneficial effects of treatment with AG on myocardial contractile function. Further studies are needed to investigate the mechanism of iNOS effects on cardiac contractility in hemorrhagic shock.
